# SFU-store-nav: A multimodal dataset for indoor human navigation

**DOI:** 10.1016/j.dib.2020.106539

**Published:** 2020-11-18

**Authors:** Zhitian Zhang, Jimin Rhim, Mahdi TaherAhmadi, Kefan Yang, Angelica Lim, Mo Chen

**Affiliations:** School of Computing Science, Simon Fraser University, Burnaby, BC, Canada

**Keywords:** Multimodal, Intent inference, Trajectory prediction, Motion tracking

## Abstract

This article describes a dataset collected in a set of experiments that involves human participants and a robot. The set of experiments was conducted in the computing science robotics lab in Simon Fraser University, Burnaby, BC, Canada, and its aim is to gather data containing common gestures, movements, and other behaviours that may indicate humans’ navigational intent relevant for autonomous robot navigation. The experiment simulates a shopping scenario where human participants come in to pick up items from his/her shopping list and interact with a Pepper robot that is programmed to help the human participant. We collected visual data and motion capture data from 108 human participants. The visual data contains live recordings of the experiments and the motion capture data contains the position and orientation of the human participants in world coordinates. This dataset could be valuable for researchers in the robotics, machine learning and computer vision community.

## Specifications Table

**Table:** 

Subject	Computer Vision and Pattern Recognition
Specific subject area	Human Intent Inference
Type of data	Video data, in AVI file format. Vicon motion tracking data, in CSV file format.
How data were acquired	Hardware: Logitech Webcams Vicon Motion Capture System Softbank Pepper robot Software: Robot Operating System [1] Python
Data format	Extracted Processed
Parameters for data collection	The data collection experiments were conducted under two conditions: one-participant and two-participant. For one-participant trial, a single participant performs the experimental task by himself/herself. And for the two-participant trial, each of the two participants perform the experimental tasks in the physical presence of the other.
Description of data collection	The data was collected in an indoor lab space set up mimic a shopping scenario. Four webcams were placed at the corners of the lab to capture the visual data in the room. A Pepper robot was placed in the lab to interact with the participants and record visual data through its own camera. The participants were asked to wear a helmet with motion capture markers on it so that the Vicon motion capture system could track their positions and head orientation.
Data source location	Institution: Simon Fraser University City/Town/Region: Burnaby, British Columbia Country: Canada
Data accessibility	https://www.rosielab.ca/datasets/sfu-store-nav
Related research article	Author's name: Zhitian Zhang Title: Towards a Multimodal and Context-Aware Framework for Human Navigational Intent Inference Conference: The 22nd ACM International Conference on Multimodal Interaction (ICMI2020) DOI: 10.1145/3,382,507.3421156

## Value of the Data

• Current human-robot interaction datasets are very limited in terms of data modality. Our dataset contains not only the visual information collected from the experiments, but also important information such as participants’ positions and head orientations.
• Researchers in computer vision, machine learning, affective computing and robotics can all benefit from this dataset. In robotics research, this data can be used to infer human navigational intent and help create better and safer robot planning algorithms. Computer vision researchers can use the dataset for topics like trajectory prediction, human body pose estimation, etc. and benefit from our multi-camera setup.
• Compared to other currently available human movement dataset [2], [3], [4], [5], our dataset captured human's ground truth location in real-world coordinates, in addition to providing visual information. Our data from two-participant trials may also capture possible useful information on human-human interactions when a robot is present.
• In our data collection experiment, we purposely designed a scenario where the human participants will become confused and ask robot for help. Such scenarios, with a realistic shopping environment involving confused humans and robot that are ready to assist humans, are valuable to study.

## 1 Data Description

### 1.1 Raw data

Raw data is captured through the Robot Operating System (ROS) and stored in ROS bag files. Raw data can be extracted into visual data, human position data and human head orientation data.

### 1.2 Visual data

**Fig. 1:**
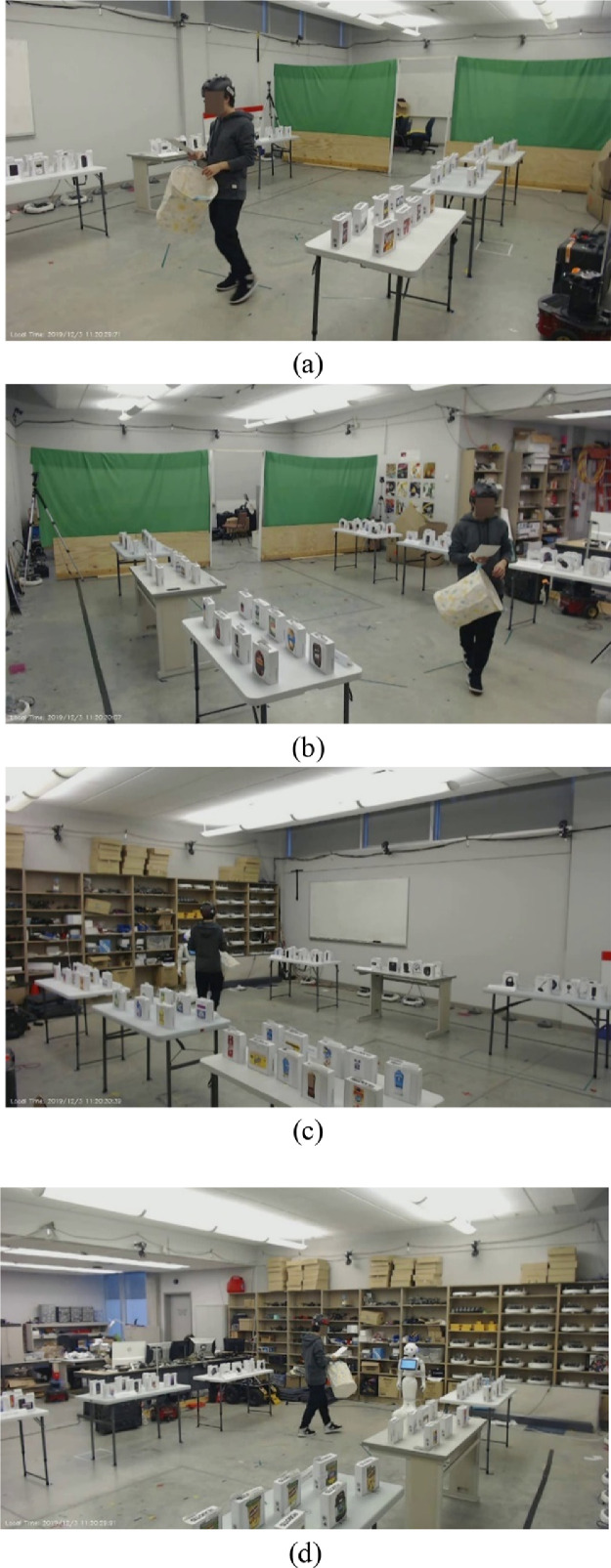
Anonymized images captured from four webcams.

There are two sources of visual data: webcams and Pepper robot's built-in camera. We record the experiment with four webcams and Pepper robot's camera. Fig. 1 shows the images that are captured through webcams at the same moment. However, due to the low resolution of Pepper robot's camera, only videos recorded from webcams are included in this dataset. Images are extracted from the ROS bag files and compressed into video files using ROS and OpenCV [6]. Every frame in the video are labelled with the time, and we also provide a CSV file that records total frame numbers and their corresponding time stamps for each video. The resolution of the video captured from webcams is 1280 × 720 pixels. To protect the privacy of the participants that involves in the experiment, we used an open source tool [7] to anonymize videos captured from webcams as shown in Fig. 1.

### 1.3 Human position and head orientation data

**Fig. 2:**
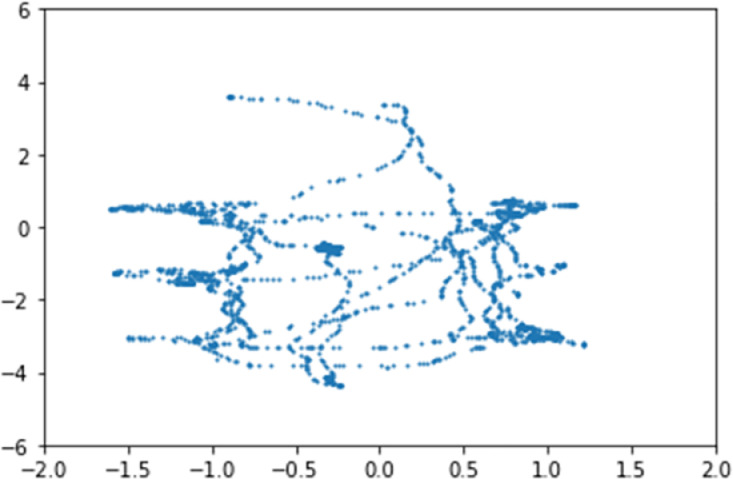
Human position data captured from Vicon system.

Participants’ real time position data and head orientation data are obtained through a Vicon motion capture system using the ROS framework. Both position and head orientation are in real world coordinates and stored in CSV files. Each position/head orientation data point corresponds to a set of images with the same time stamp. That means our position/head orientation data are synchronized with the image data in time. Position data is described in (X, Y) format and head orientation is described in (roll, pitch, yaw) format. Fig. 2 shows the position data for a whole experiment trial.

## 2 Experimental Design, Materials and Methods

The aim of the experiment is to gather data containing common gestures that may indicate humans’ navigational intent relevant for autonomous robotic navigation. The experiment simulated shopping in an electronic store and a grocery store, an activity that encourages natural human movements and provides an opportunity to interact with a robot.  In real life, people do not always shop alone. Moreover, different shopping experiences (e.g., shopping alone, shopping in a familiar or unfamiliar environment) would lead to different navigation patterns. Therefore, we created two study conditions: one-participant trial and two-participants trail. Details of the study conditions are described below:

1. One-participant trial:
  a A single participant performs the study tasks in the presence of a robot, for the first time, as a “newcomer” of an unfamiliar environment.
  b This participant will repeat the study tasks, with a different shopping list, as an “oldcomer” who is familiar with the store environment.
2. Two-participants trial: Two participants conduct study tasks in the physical presence of another. One participant is a newcomer conducting the task for the first time, and the other participant will be an oldcomer who has conducted the task before. They will also conduct the study task in the presence of a robot.

### 2.1 Participants

The study was conducted in Simon Fraser University, Burnaby, BC, Canada. There were 108 participants total, and each received a $10 CAD gift card. Participants were recruited via class announcements and school mailing lists. There are 36 one-participant trials and 36 two-participants trial in total. Although the number of trials is the same, the number of participants involved in the one-participant trials is significantly higher than those involved in two-participant trials.

### 2.2 Materials and experimental set up

**Fig. 3:**
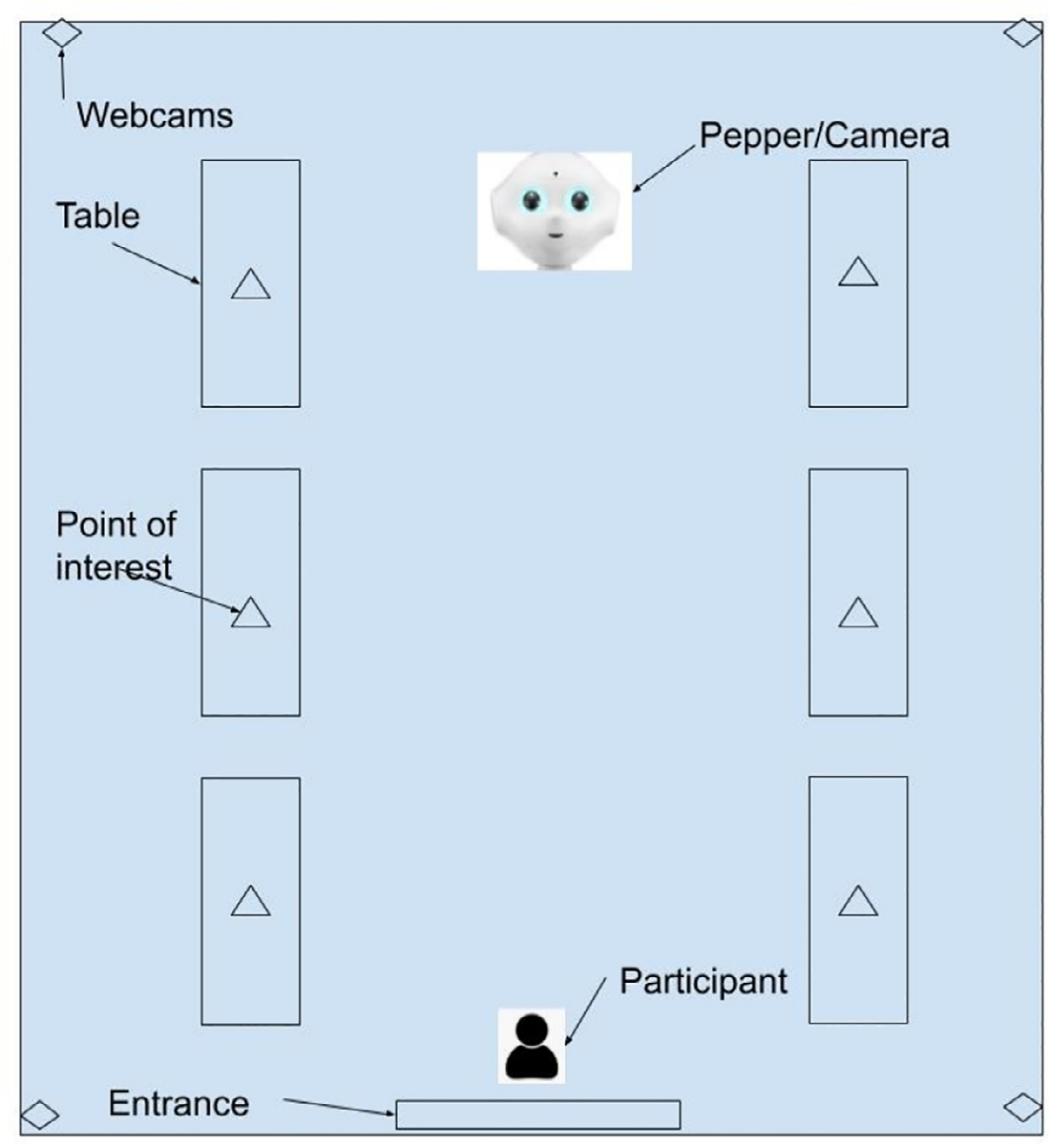
Experiment Set up.

We used a Pepper robot by Softbank robotics, two computers, and a Vicon motion capture system in this experiment. Pepper was used because it is a programmable robot which is designed to interact with human users, which allows the data to be representative of how humans may behave while interacting with a robot. The robot was programmed using NAOqi SDK,^1^ a Python based development tool for the Pepper. One computer was used by the Pepper operator to coordinate Pepper's dialogues, which was pre-programmed to give the illusion that the robot is autonomously responding to the participants. The other computer was used to record the frequency and duration of participants’ interaction with the robot. A total of five cameras recorded the participants’ interactions with Pepper.  Four web cameras were placed at each corner of the experiment room to capture full movements of participants, and the built-in camera of Pepper captured human behaviors from the Pepper's perspective. To gather data for the Vicon motion capture system, we placed reflective markers on a biking helmet which each participant is instructed to wear during the experiments. For two-participant trials, the reflective markers were placed in unique patterns to distinguish two users. A sample experiment setup layout is shown in Fig. 3.

## 3 Procedure

Participants signed a consent form before beginning the task. If they agreed to participate in the study, we provided a brief overview of the research and provided study protocol. The participants were provided with 4 to 8 different shopping lists and were asked to find all the items on the provided list. Each list had six items and one or two items were intentionally missing to encourage the participant to interact with the robot. Participants were advised to ask the robot for help if needed when finding items. During the experiments, participants were asked to wear the biking helmet with Vicon markers throughout the task so that their position and head orientation can be measured by the Vicon motion capture system. The study was conducted using a partial Wizard of Oz method. As the robot was not fully autonomous, when participants approached and asked questions to the robot, researcher played pre-recorded scripts of the robot to answer questions. Some examples of the scripts that are used for robots are listed below:

• How can I help you today?
• That's a great question, let me check it out for you.
• The item you are looking for is on Table A.
• Sorry the item you are looking for is out of stock.

## Ethics Statement

The study was approved by SFU ethics board (Study number: 2019S027). We confirm that informed consent was obtained for experimentation with human participants.

## Declaration of Competing Interest

The authors declare that they have no known competing financial interests or personal relationships which have, or could be perceived to have, influenced the work reported in this article.

## Footnotes

1 http://doc.aldebaran.com/2-4/dev/python/index.html

## References

[1] Quigley M., Conley K., Gerkey B., Faust J., Foote T., Leibs J., Wheeler R., Ng A.Y. (2009).

[2] Robicquet A., Sadeghian A., Alahi A., Savarese S. (2016). Learning social etiquette: human trajectory understanding in crowded scenes. European Conference On Computer Vision.

[3] Linou K. (2016). Nba-player-movements. https://github.com/linouk23/NBA-Player-Movements.

[4] Pellegrini S., Ess A., Schindler K., Van Gool L. (2009). You'll never walk alone: modeling social behavior for multi-target tracking. Proceedings of the 12th IEEE International Conference on Computer Vision.

[5] Lerner A., Chrysanthou Y., Lischinski D. (2007). Crowds by example.

[6] Bradski G. (2000). The OpenCV Library.

[7] Li J., Wang Y., Wang C., Tai Y., Qian J., Yang J., Wang C., Li J., Huang F. (2019). DSFD: dual shot face detector. Proc. IEEE Comput. Soc. Conf. Comput. Vis. Pattern Recognit.

